# Phenotypic and genetic characterisation of an emerging reovirus from Pekin ducks in China

**DOI:** 10.1038/s41598-019-44178-3

**Published:** 2019-05-23

**Authors:** Yanxin Cao, Mengxu Sun, Jun Wang, Xueying Hu, Weiyong He, Jingliang Su

**Affiliations:** 1 Key Laboratory of Animal Epidemiology and Zoonosis, the Ministry of Agriculture, College of Veterinary Medicine, China Agricultural University, Beijing, 100193 China; 2 College of Veterinary Medicine, Huazhong Agricultural University, Wuhan, 430070 China

**Keywords:** Viral pathogenesis, Viral epidemiology, Viral pathogenesis, Viral epidemiology

## Abstract

In June 2016, a disease characterised by intestinal haemorrhage with a mortality rate of approximately 5% was observed in a duck farm in Shandong province, China. Here, we report the isolation and characterisation of a reovirus from duck tissue samples by inoculating duck embryos and duck embryo fibroblasts (DEF). The isolate replicated in DEF and Vero cells and formed syncytia. Sequence analysis revealed that the viral genome was 23,434 nt in length with typical structure organization, consisting of 10 dsRNA segments ranging from 3998 nt (L1) to 1190 nt (S4) in size, and was genetically distinct from previous Chinese duck-origin reoviruses. Phylogenetic analyses showed that the isolate was most closely related to the recently reported duck reovirus D2533/6/1-10 isolated in Germany, forming a monophyletic branch different from known reference avian reoviruses. Experimental infection results indicated that the isolate replicated transiently in ducklings and was shed via faeces. Infection with the isolate caused epithelial cell damage and lymphocyte apoptotic death in the bursa of Fabricius, which may result in immunosuppression in infected ducklings. The role of the isolate in current duck haemorrhage enteritis remains to be determined, but its damage to the bursa warrants further investigation of the duck immune response.

## Introduction

Avian reoviruses (ARVs) are widely distributed in a variety of avian species, including wild birds^1–4^. They are members of genus *Orthoreovirus* in the family *Reoviridae*. The viral double stranded RNA genome contains 10 segments divided into three size classes based on their electrophoretic motility on a sodium dodecyl sulfate-polyacrylamide gel: large (L1, L2 and L3), medium (M1, M2 and M3) and small (S1–S4)^5^. The genome is predicted to encode eight structural proteins (λA, λB, λC, μA, μB, σA, σB and σC) and several nonstructural proteins (μNS, σNS, p10, etc). ARVs have been associated with a variety of diseases in domestic fowl, including chickens (viral arthritis/tenosynovitis, malabsorption syndrome, gastroenteritis and respiratory disease)^6^, turkeys (infectious enteritis)^7^, ducks and geese (fatal infection and spleen necrosis)^8–10^. In wild birds, reoviruses have been detected in white stork, grey heron, rock pigeon and so on^4^. ARV isolates were traditionally classified using standard serological procedures, but more recently have been characterised by molecular methods based on genome sequences and deduced amino acid analyses. However, for pathogenic isolates, it is difficult to assign a new isolate to a pathogenic group or pathotype according to its genetic background due to the lack of clearly defined virulence determinant(s) of the avian reovirus.

In China, Muscovy duck reovirus (MDRV) infection was first recognised in domestic Muscovy ducks in 2001^11^, and the disease was seen only in Muscovy ducks (*Carina moschata*) and mule ducks, an infertile hybrid cross between male Muscovy and female Pekin ducks (*Anas platyrhynchos*). Genetic analysis showed that the virus was closely related to MDRV isolated in Europe^12^. Since 2006, outbreaks of disease characterised by spleen necrosis in Pekin ducklings were observed, and the aetiological agent was identified to be a reovirus distinct from previous MDRV isolates^10^. Currently, the reovirus has been tentatively classified as a “novel” duck reovirus (NDRV) to distinguish it from the “classical” MDRV^13,14^. Virus infections were subsequently diagnosed widely in mainland China, affecting almost all domestic ducks and geese^13,15–17^. In June 2016, an outbreak of disease characterised by intestinal haemorrhage was observed in a commercial duck farm in Shandong Province, with a mortality rate of around 5% before the age of 4 weeks. In this study, we conducted an aetiological investigation and isolated a reovirus genetically distinct from previous duck reoviruses identified in China. The pathogenicity of the isolate was evaluated in ducklings.

## Results

### Virus isolation and characterisation

**Figure 1:**
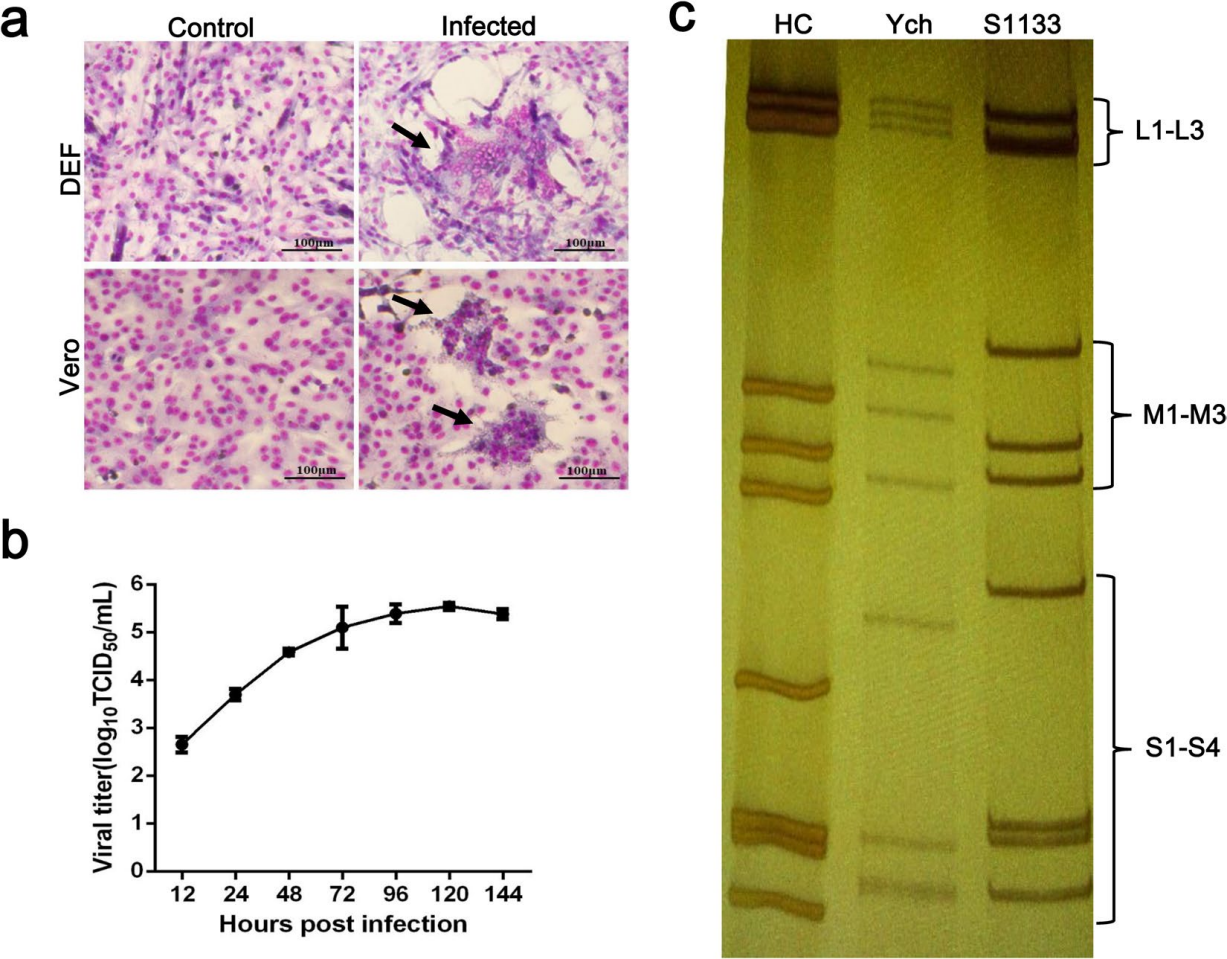
Characteristics of the isolated Ych strain. (**a**) Syncytium formation induced by Ych in DEF and Vero cells at 48 hours-post-infection (Giemsa stain). (**b**) Growth kinetics of Ych in DEF cells. Cells were infected with virus at a multiplicity of infection of 0.01. The titers represent the means ± SD (n = 3) from one of the three independent experiments. (**c**) SDS-PAGE analysis of the genomic segment mobility of HC (a duck reovirus), Ych and S1133 (a chicken reovirus). Full-length gel is presented in Supplementary Fig. S1.

Bacterial infection was first ruled out since no typical pathogenic bacterium was isolated from tissue samples. Attempts were then made for virus isolation. The mixed tissue homogenate was inoculated into duck embryos and sequentially passaged eight times. No consistent mortality was observed for inoculated embryos, but irregular necrotic foci were found in the livers and spleens of some embryos at necropsy on 5 dpi. We then infected DEFs with embryo allantoic fluid of the fourth passage and observable CPE, characterised by the formation of a few syncytia and cell detachment appeared on 4 dpi after four successive passages. Further passage in DEFs resulted in the rapid formation of syncytia and the virus titre peaked at 3.47 × 10^5^ TCID_50_/mL (Fig. 1a,b). Thus, the isolate was designated as Pekin duck/China/Ych/2016 (Ych hereafter). When DEF-adapted Ych was used to infect fresh Vero cell monolayers, syncytium formation was observed (Fig. 1a), suggesting the isolate replicated in Vero cells. Infectious virus quantitation in cell culture showed that Ych was resistant to chloroform treatment and virus replication in DEFs was not significantly affected by addition of 5-bromo-2′-deoxyuridine in the maintenance medium. These results indicated that the isolate was a nonenveloped RNA virus.

To identify the isolate, genomic RNA was extracted from concentrated virus particles and constructed RNA library was subjected to next generation sequencing (NGS). Analysis of the NGS data revealed that Ych was a reovirus distinct from the known China-origin duck reovirus isolates. To confirm the isolate, we designed a primer pair specific for the Ych λC gene on the basis of the NGS sequence data, and reverse transcription-polymerase chain reaction (RT-PCR) was performed using the RNA template extracted from the original duck tissue sample. A gene fragment identical to that revealed by deep sequencing was obtained. We further analysed the electrophoretic mobilities of the viral double-strand RNA segments and revealed that the ten genomic segments displayed clearly distinguishable migration patterns from those of duck reovirus DRV-HC and ARV-S1133 (Fig. 1c). It was noticed that the S3 segment of Ych migrated more closely to S4 rather than the S2 segment in comparison with ARV-S1133 and DRV-HC. Differences were also evident in other segments with the exception of L1–L3.

### Genomic analysis of the Ych isolate

Based on the NGS data, a draft genome sequence was first aligned and then verified by Sanger sequencing using designed primer pairs (Table 1). The resulting genome sequence of Ych was 23,434 nucleotides (nt) in length, and the genome organisation was similar to reference ARVs, consisting of 10 segments from 1190 nt (S4) to 3998 nt (L1) (GenBank accession numbers MK173029–MK173038) (Table 2). The 5′ untranslated region (UTR) of the positive-sense strand ranged in length from 12 to 30 nt, and the 3′-UTR ranged in length from 32 to 98 nt, containing the same 5′and 3′terminal nucleotide motifs (5′-GCUUUU…UAUUCAUC-3′) as described for other avian reoviruses^16,18^. Proteins encoded by representative avian reovirus homologous segments were identified in the genome (Table 2). The polycistronic S1 segment was predicted to encode putative proteins p10, p13 and σC, homologous to chicken ARV p10, p17 and σC, respectively^19^.

**Table 1:** Primers used for amplification and sequencing of the isolate Ych.

Gene segment	Primer name	Sequence (5′ → 3′)	Location	Product size (bp)
S1	Ych-S1F	GCATGCAATGGTGGTACAGTG	35–55	1506
	Ych-S1R	CTTACTGCGTGACATGGACC	1521–1540	
S2	Ych-S2F	GTACGAGTTTTTCTCTGTGCC	30–50	1238
	Ych-S2R	CCTAATTGGTGAAAGTGGCC	1248–1267	
S3	Ych-S3F	CAATGGAGGTGCGTATGCC	29–47	1049
	Ych-S3R	CTGAAGGTAGTGGGTCGTGTC	1057–1077	
S4	Ych-S4F	CAACACTTCTGCTGCTGCCG	56–75	1055
	Ych-S4R	GGGAAACAGACAATAAGACG	1091–1110	
M1	Ych-M1-1F	CTATCTAGCCACACCCGTG	18–36	1313
	Ych-M1-1R	CGTCACTATCCATAATAGTG	1311–1330	
	Ych-M1-2F	GATATCAGATGCTTCGGGAAG	1074–1094	965
	Ych-M1-2R	CAGCTACGATGCGAAATTCG	2019–2038	
M2	Ych-M2-1F	TATCGCTCACCATGGGCAAC	20–39	1187
	Ych-M2-1R	CTCATTCGGATTGAACGAGCC	1186–1206	
	Ych-M2-2F	CCTCGCACTTACAACATCCG	973–992	1145
	Ych-M2-2R	CTGGCGTGGATTCAGCTTAAC	2097–2117	
M3	Ych-M3-1F	ATGATGGCGTCCACTAAGTGG	22–42	1388
	Ych-M3-1R	CGATTCATACGTTGCAGATCC	1389–1409	
	Ych-M3-2F	CAGATATGGTAGCGTGTCGAC	1242–1262	730
	Ych-M3-2R	CGTCCATGATCCACGTTGAG	1952–1971	
L1	Ych-L1-1F	GCTCCAGTTTCTGAGAAGAAAG	60–81	1249
	Ych-L1-1R	CTCTCGAGGGATACATGACC	1289–1308	
	Ych-L1-2F	TCTTGAGCAACTTGCACCTC	1165–1184	1292
	Ych-L1-2R	CATCGACGCTCTAATCGATTG	2436–2456	
	Ych-L1-3F	CCTATACGGATCACTAATCCG	2316–2336	1589
	Ych-L1-3R	CAATCGATTAGAGCGTCGATG	3885–3904	
L2	Ych-L2-1F	CAGTCAAAGGTGTTTTGGCC	19–38	1337
	Ych-L2-1R	CTGAAATAGAGGGTCCCAAG	1336–1355	
	Ych-L2-2F	CACCCTATTGGTTCCTTACG	1227–1246	1336
	Ych-L2-2R	CTGAATAGGCTAGAGAAAGC	2543–2562	
	Ych-L2-3F	GTGATCGCTTGGAGATGTGG	2434–2453	1324
	Ych-L2-3R	CGCACAAAGTTCTGCATTCC	3738–3757	
L3	Ych-L3-1F	CAGATTCGAGGTTTGCGCTTG	19–39	1344
	Ych-L3-1R	CAAGTCCAATGGATAACCAGC	1342–1362	
	Ych-L3-2F	ATTCCCTTTGCTGGCATGC	1218–1236	1400
	Ych-L3-2R	GGTAGTCCAACTGCATGTAG	2598–2617	
	Ych-L3-3F	CTTCCACTGGCTGGATTGTG	2466–2485	1375
	Ych-L3-3R	TGGAGGCACGTAGAAAGACG	3821–3840

**Table 2:** Genetic characteristics of duck reovirus strain Ych.

Genome segment	Size (nt)	Length (nt) of			Encoded protein and size (aa)	Strain with highest amino acid similarity (%)
		5′UTR	ORF	3′UTR		
L1	3998	20	3921	57	λA (1306)	D2533/6/1-10 (98.1)
L2	3826	14	3780	32	λB (1259)	D2533/6/1-10 (97.4)
L3	3899	12	3855	32	λC (1284)	D2533/6/1-10 (94.9)
M1	2282	12	2199	71	μA (732)	D2533/6/1-10 (93.3)
M2	2150	30	2022	98	μB (673)	D2533/6/1-10 (98.5)
M3	1990	21	1908	61	μNS (635)	D2533/6/1-10 (95.8)
S1	1573	22	282	34	p10 (93)	D2533/6/1-10 (94.7)
			369		p13 (122)	D2533/6/1-10 (92.7)
			1014		σC (337)	D2533/6/1-10 (91.7)
S2	1325	15	1251	59	σA (416)	D2533/6/1-10 (96.4)
S3	1201	30	1104	67	σB (367)	D2533/6/1-10 (97.6)
S4	1190	23	1104	63	σNS (367)	D2533/6/1-10 (97.0)

Nucleotide and amino acid (aa) sequences of all segments of Ych were compared with representative members of *Orthoreovirus* species (Table 3 & Tables S1–S10 in Supplementary Materials). Moderate sequence identities were seen for the relatively conserved fragments encoding inner core proteins of ARVs, including the λA encoding gene (71.5–72.7% nt, 83.8–84.5% aa), λB (66.0–67.4% nt, 75.5–76.1% aa), λC (55.7–56.5% nt, 55.4–56.0% aa), μA (58.4–60.7% nt, 60.4–61.1% aa) and σA (63.6–64.3% nt, 66.7–67.4% aa). In terms of more divergent outer capsid proteins, Ych shared sequence identity values with those of the representative isolates of ARVs: μB (62.5–65.9% nt, 66.5–72.1% aa), σB (55.8–61.2% nt, 52.4–60.3% aa), and σC (37.3–43.6% nt, 22.2–26.7% aa). The nt and aa identity could not absolutely satisfy the species demarcation criteria in the genus *Orthorovirus*^18^, but fell into the grey zone of the cut-off values, with the exception of σC. Interestingly, Ych shared the highest nt/aa identity with the newly-described reovirus D2533/6/1-10 genome segments at a level ranging from 85.5–94.1% (nt) and 91.7–98.5% (aa), which was isolated from Pekin ducklings in Germany^20^.

**Table 3:** Comparison of nucleotide and amino acid sequence identities of Ych with representative members of *Orthoreovirus* species (%).

Strains		λA	λB	λC	μA	μB	μNS	σC	σA	σB	σNS
ARV-D2533/6/1-10	nt	94.1	91.7	87.7	93.4	89.6	92.1	85.5	89.6	91.8	90.3
	aa	98.1	97.4	94.9	93.3	98.5	95.8	91.7	96.4	97.6	97.0
ARV-Ch/Tu^a^	nt	72.0–72.8	66.6–67.3	55.2–56.1	59.4–60.3	64.2–65.7	57.6–58.9	33.6–37.0	63.6–64.7	54.8–58.7	61.2–62.9
	aa	84.3–85.0	75.4–76.3	54.2–55.2	60.4–61.7	69.6–72.4	55.8–57.6	21.6–24.4	66.7–67.6	51.9–55.7	62.8–64.9
ARV-Wa Classical^b^	nt	71.3–72.3	65.8–66.5	56.0–56.4	57.6–60.6	62.4–62.7	57.7–58.5	36.0–38.0	63.3–64.8	59.1–60.2	61.3–62.7
	aa	83.8–84.2	75.5–76.4	55.3–55.6	60.0–61.1	66.5–66.9	55.8–57.4	24.1–25.2	66.2–67.1	55.2–57.6	63.3–64.9
ARV-Wa Novel^c^	nt	71.4–72.1	65.8–66.3	56.0–56.4	60.3–60.7	65.7–66.0	57.3–57.8	39.6–44.5	63.8–64.9	60.6–61.6	60.7–62.0
	aa	83.8–84.6	75.6–75.9	55.8–56.0	60.7–61.3	71.2–72.0	55.5–56.6	26.7–27.3	66.7–67.4	59.2–60.6	63.0–64.1
NBV^d^	nt	65.3–66.0	36.5–56.1	45.8–46.7	49.5–50.9	62.7–63.1	28.4–48.4	25.8–31.1	56.8–58.1	43.2–44.2	55.2–55.4
	aa	72.2–72.6	36.7–40.1	36.4–40.8	45.3–46.2	68.3–69.2	7.6–38.3	6.5–17.7	56.1–56.6	30.0–30.3	51.1–51.6
BRV^e^	nt	36.7	53.2	39.8	44	47.6	39.8	—	41.6	35.4	41.4
	aa	8.4	49.4	27.9	31.9	39	25.8	—	29.9	16.9	25.6
RRV^f^	nt	54.4	55.3	40.7	46.6	56.5	39.5	34.2	42.9	39.5–39.9	44
	aa	49.7	56.3	28	38.7	53	24.8	16.1	33.8	20.4–21.3	31
MRV^g^	nt	51.0–51.3	53.9–54.6	41.0–41.8	40.9–41.4	50.4–51.0	38.4–40.1	25.3–28.5	41.8–43.5	35.4–36.4	40.4–41.4
	aa	42.9–43.5	52.2–52.7	28.2–28.6	26.8–27.4	44.9–45.7	20.9–21.7	11.6–15.4	27.2–28.4	14.7–16.4	23.1–24.0

^a^Chicken (S1133, 138, 176, AVS-B, GX/2010/1, T1781) and turkey (19831M09, 22342/13, D1246) origin ARVs.

^b^“Classical” waterfowl origin ARVs: Muscovy duck (ZJ2000M, 815-12, D1546, D2044) and goose (D20/99).

^c^“Novel” waterfowl origin ARVs: Muscovy duck (ZJ00M, NP03, J18), Pekin duck (TH11, HC, 091), wild Mallard duck (SD-12) and goose (03G).

^d^NBVs (AF059718.1, AF059722.1, AF059726.1, AF218360.1, JF342672.1-JF342677.1, JF342666.1-JF342671.1, AY357730.1-AY357733.3, JF811580.1-JF81153.1).

^e^BRVs (AF059719.1, AF059723.1, AF059727.1, HQ847903.1-HQ847908.1).

^f^RRVs (AY238886.1, KT696547.1-KT696556.1).

^g^MRVs: MRV-1 (B/03), MRV-2 (BYD1), MRV-3 (T3D, ZJ2013), MRV-4 (Ndelle).

“—”: no equivalent sequence.

**Figure 2:**
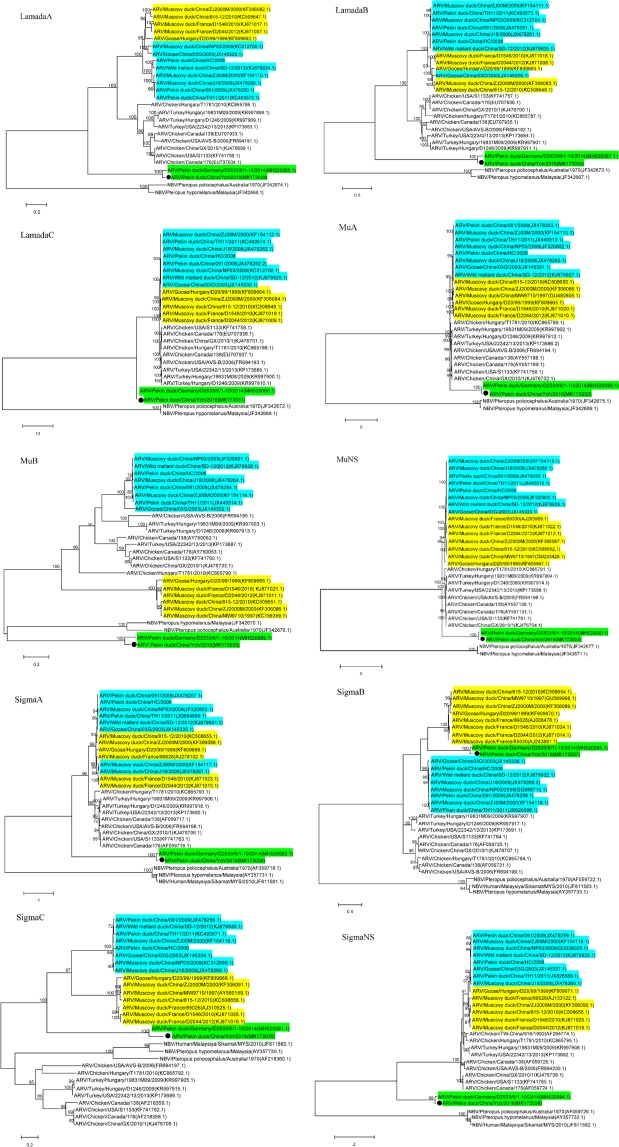
Phylogenetic relationship of Ych to selected *orthoreovirus* species based on the nucleotide sequences of ten ORFs. Maximum likelihood trees were constructed using a General Time Reversible model (MEGA 7.0.14 program) with bootstrap values calculated from 1000 replicates. Bootstrap values lower than 0.7 were hidden. GenBank accession numbers of reference strains appear next to the virus names. The “classical” waterfowl origin ARVs, “novel” waterfowl origin ARVs and newly emergent duck reovirus are marked with yellow, blue and green backgrounds, respectively.

The open reading frames (ORFs)of the nucleotide sequences of ten segments were used to construct the phylogenetic trees (Fig. 2). Phylogenetic analyses revealed a strong evolutionary relationship with strain D2533/6/1-10, and Ych always appeared on the same monophyletic branch with the German isolate, which was distinct from other known orthoreovirus isolates. Based on our analyses, the waterfowl-origin reoviruses could be divided into three genogroups, (i) the “classical” Muscovy duck reovirus strains isolated from Muscovy ducks and geese, (ii) the “novel” duck reovirus strains, (iii) the newly-emerging duck reovirus strains Ych and D2533/6/1-10.

### Serological characterisation

The serotype relationship of Ych with duck reovirus DRV-HC and ARV-S1133 was determined by virus neutralisation test (Table 4). The three strains were neutralised by homologous antiserum, however, they could not be cross neutralised by antibody against a heterologous strain. The result demonstrated that Ych was serologically different from DRV-HC and S1133.

**Table 4:** Serological relationships between Pekin duck and chicken origin reoviruses studied by cross-neutralisation tests.

Virus	Neutralisation titre of antisera to^*^		
	Ych	HC	S1133
Ych^a^	4	<1	<1
HC^a^	<1	5	<1
S1133^b^	<1	<1	4

*(−log2).

^a^Pekin duck origin ARVs.

^b^Chicken origin ARV.

### Results of experimental infection

Compared with uninfected controls, no abnormal clinical signs were noted in ducklings infected by oral/intranasal or subcutaneous inoculation during the experimental period, and neutralising antibody was not detected in sera collected from the infected ducks on 9, 12 and 16 dpi. However, virus-specific RNA could be sequentially detected from cloacal swabs of some infected ducklings from 1–4 dpi (Table 5), suggesting the virus replicated in ducklings. As 12-day-old ducklings were infected by simultaneous oral and subcutaneous inoculation, virus specific RNA was detected from caecal tonsil (1/5) and bursa of Fabricius (3/5) when ducklings were euthanised on 3 dpi. Virus could also be recovered after inoculation of the RT-PCR-positive samples into DEF monolayers. These data further indicate that the isolate replicated in the intestinal lymphoid tissue and bursa of Fabricius of infected ducklings. However, the virus was not detected in tissue samples collected on 5 dpi and thereafter, suggesting that the virus infection of the bursa was transient.

**Table 5:** RT-PCR detection of reovirus Ych virus gene in cloacal swab samples from experimentally infected ducklings.

Days post-infection	Infection route		
	Oral and intranasal (n = 8)	Subcutaneous (n = 7)	Mock-infected control (n = 5)
1	5/8	0/7	0/5
2	8/8	0/7	0/5
3	8/8	2/7	0/5
4	2/8	5/7	0/5
5	0/8	0/7	0/5
6	0/8	0/7	0/5

**Figure 3:**
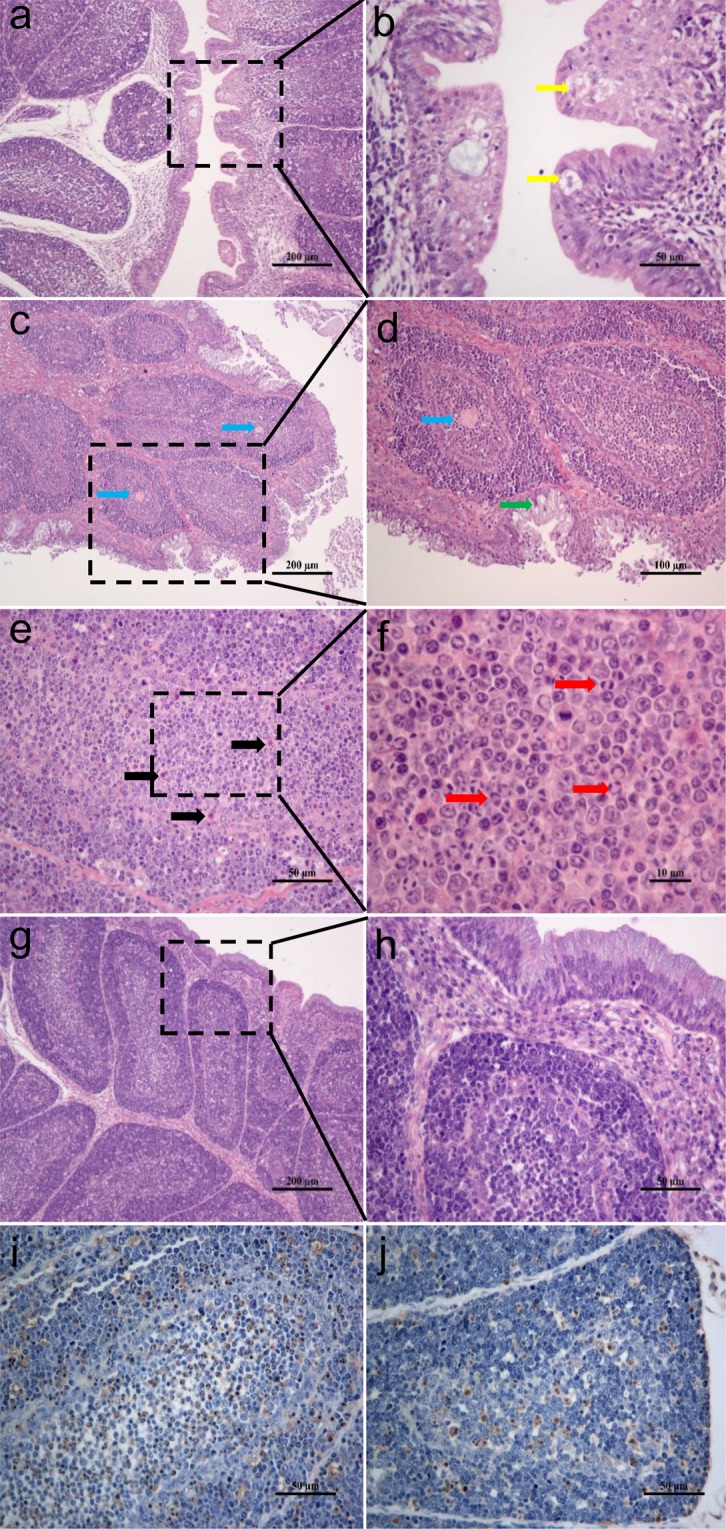
Microscopic lesions in the bursa of ducklings infected with reovirus Ych isolate. (**a**,**b**) showing the inter-follicle edema, vacuolation of epithelial cells (yellow arrow) (HE stain) (Scale bar = 200 & 50 μm, respectively). (**c**,**d**) showing the epithelial cell sloughing, goblet cell metaplasia in the mucosal layer (green arrow) and central eosinophilic focus (blue arrow) (HE stain) (Scale bar = 200 & 100 μm, respectively). (**e**,**f**) showing monocyte infiltration (black arrow) and cells with chromatin margination (red arrow) (HE stain) (Scale bar = 50 & 10 μm, respectively). (**g**,**h**) showing bursa sections of uninfected ducklings (HE stain) (Scale bar = 200 & 50 μm, respectively). (**i**,**j**) Marking of apoptotic cells of one infected duckling at 5 dpi (**i**) and uninfected control (**j**) with TUNEL assay. Haematoxylin stain was used as a cytoplasmatic contrast (Scale bar = 50 μm).

Histopathological examination of the tissues of infected ducklings revealed inter-follicle oedema of the bursa of Fabricius (Fig. 3a). The epithelial cells of the bursa underwent vacuolar degeneration and debris or mucin were seen inside the vesicles on 3 dpi (Fig. 3b). On 5 dpi, marked sloughing of epithelial cells and apparent goblet cell metaplasia were seen in the mucosal layer (Fig. 3c,d). Monocyte infiltration and central eosinophilic focus formation were observed in the medullar area of the bursa follicles (Fig. 3c,d,e). Lymphocytes exhibited apoptotic images characterised by nuclei margination (Fig. 3f). No abnormality was observed in bursa of uninfected ducklings (Fig. 3g,h).TUNEL assay revealed an increase in the number of apoptotic cells compared with the mock control (Fig. 3i,j). These results suggested that Ych infection could induce damage to the bursa of Fabricius of infected ducklings although viral replication persisted for a short period only.

## Discussion

ARVs have been demonstrated to be involved in numerous diseases in commercial poultry and the pathogenicity of isolates differs considerably. Differences in pathogenicity have been described for a number of avian reoviruses^6,7,21,22^, and high morbidity and mortality with multifocal hepatic and spleen necrosis at necropsy have been recorded for the majority of duck and goose reovirus infections^9–11,13,15,23–25^. In this work, a reovirus Ych was isolated from tissue samples of diseased Pekin ducks, characterised by intestinal haemorrhage. The isolate could induce syncytia formation in infected cell cultures but did not cause duck embryo death as previously described for pathogenic duck reoviruses. Genomic analyses showed that Ych shared the common genome structural characteristics and the predicted polycistronic S1 genome segment, but it was genetically distant from known reovirus isolates identified in China^10^. It is interesting that the greatest sequence similarity values of the ten genome segments were seen with the recently described reovirus strain D2533/6/1-10, which was isolated from a bursa sample of Pekin ducks in Germany^20^. Phylogenetic analysis of all genes revealed that the two isolates form a distinct branch from reference ARVs, suggesting that they might have a common evolutionary origin. Based on the relatively small amount of ARV sequence data available in GenBank, no attributable genomic reassortment or intra-segment recombination was found for the evolutionary origin of the newly emergent duck reovirus. Cross neutralisation results demonstrated that Ych belongs to a serotype distinct from the “novel” duck reovirus and chicken reovirus S1133.

In this study, inoculating ducklings with Ych by oral, intranasal or subcutaneous routes, which are likely natural transmission routes for avian reoviruses, did not produce clinical disease and gross lesions, as were observed in the field cases. However, virus shedding from the infected ducklings was detected by RT-PCR, suggesting that the virus multiplied transiently in the infected duck intestine. However, virus shedding via faeces implies that infected ducklings may transmit virus laterally to pen mates as seen in other reoviruses in chickens and turkeys^26^. It was noticed that the German strain D2533/6/1-10 was isolated from a sample of duck bursa although its pathogenicity was not further evaluated^20^. Our experimental infection results demonstrate that the virus replicated in the bursa of Fabricius. These findings are also in agreement with previous reports that the intestine and bursa of Fabricius were the initial sites of replication of some chicken and turkey reovirus isolates^27,28^. Microscopic examination revealed damage to the bursa epithelial cells and increased number of apoptotic lymphocytes, suggesting that Ych infection might induce a transient and possibly permanent immunosuppression in infected ducklings. This might explain to some extent that no specific neutralising antibody against the virus was detected from the sera of infected ducklings until the end of observation period. However, the fact that virus was not detected in the spleen, liver, kidney or lung samples of the experimentally infected ducks suggests that Ych was noninvasive for these internal organs, thus affecting also the humoral immune response. This scenario has been reported in chickens for reoviruses possessing low pathogenicity^29^.

Describing the aetiological role of an ARV isolate can be complicated because some clinical disease states are often difficult to reproduce experimentally^30^. Haemorrhagic enteritis as seen in field cases was not reproduced in this study. Our experimental infection results demonstrate that the bursa of Fabricius may serve as a site of Ych replication in infected ducklings, and whether the increase of apoptotic lymphocytes could result in lymphoid cell deletion is under investigation. It was possible that under field conditions, the virus infection induced immunosuppression, predisposing the ducklings to infection with other pathogens, which would worsen the disease and lead to death.

In summary, diagnostic attempts at Pekin duck haemorrhagic enteritis led to the isolation of a new reovirus, Ych, genetically distinct and antigenically different from previous duck reovirus strains identified in China. Experimental infection of ducklings indicated that the virus was able to replicate in the bursa of Fabricius and induce epithelial cell damage and lymphoid cell apoptosis. The virus-induced damage to this immune organ implies that the newly emergent reovirus infection may serve as a priming or concurrent agent in the development of duck enteritis. However, the role of Ych in haemorrhagic enteritis needs to be further evaluated in combination with other agents or substances.

## Materials and Methods

### Virus isolation

Twenty-day-old Pekin ducks that died at a farm in Yucheng, Shandong Province were necropsied. Pathological examination revealed mild to moderate swollen liver and intestinal mucosa haemorrhagic plaques. Samples of liver, spleen and brain tissue were inoculated onto tryptic soy agar (BD, Sparks, MD, USA) plates containing 2% fetal bovine serum and incubated at 37 °C for 48 h for bacterial examination. Virus isolation was performed later. The intestinal mucus, liver and spleen samples were pooled and homogenised in sterile phosphate-buffered saline solution (pH 7.2) to give a 20% suspension (w/v). After centrifugation at 8,000 × g for 10 min, the supernatant was filtered through a 0.22 μm pore-size sterile filter and inoculated into five 9-day-old duck embryos (0.2 mL/embryo) by the chorioallantoic membrane route. Embryos were candled daily and were chilled at 4 °C to death on 5 days post-inoculation (dpi) to harvest allantoic fluid for subsequent passage. Since no consistent embryo death was observed during the serial embryo-passage, we inoculated the allantoic fluid of the fourth passage onto confluent monolayers of duck embryo fibroblasts. The syncytium formation of the infected cells was visualised by Giemsa staining. To test the growth of the isolate in Vero cells, the DEF-adapted virus suspension was inoculated onto the monolayer of Vero cells using the conventional method.

### Virus characterisation

To determine whether the isolated virus had a lipid envelope, the DEF-adapted virus suspension was clarified by low speed centrifugation after two freeze/thaw cycles and treated with chloroform (5%, v/v) for 10 min at room temperature. The chloroform layer was then removed by centrifugation and the infectious virus titre of the supernatant was quantified in DEF culture after serial dilution. For nucleic acid type determination, virus replication ability was tested in DEF culture with or without addition of 5-bromo-2′-deoxyuridine (BrdU, 50 μg/mL) in the maintenance medium. Duck Tembusu virus and duck enteritis virus were used as RNA and DNA virus controls, respectively.

### Virus RNA extraction and high-throughput sequencing

Based on the fact that the isolate was a non-enveloped RNA virus, we extracted viral RNA from the concentrated virus. Briefly, the virus-infected DEF cell suspension was centrifuged at 8,000 × g for 30 min at 4 °C to remove cell debris. The clarified supernatant was further centrifuged by ultracentrifugation at 100,000 × g for 2 h. After removing the supernatant, the pellet was resuspended in distilled water to 1/100 volume of the original supernatant and total RNA was extracted from the suspension with TRIzol (Invitrogen, Carlsbad, CA, USA). Using the extracted RNA as template, an RNA library was prepared with NEBNext^®^ Ultra^™^ RNA Library Prep Kit for Illumina (NEB, Ipswich, MA, USA) and submitted for NGS with the Illumina HiSeq4000 (Shanghai Hanyu Bio-Tech Co., Ltd, Shanghai, China).

### Genome sequence analysis

On the basis of the preliminary sequence data obtained from NGS, a set of gene-specific oligonucleotide primer pairs (Table 1) were designed. The genomic fragments were then amplified by RT-PCR and sequenced by the Sanger method to further verify the viral genomic sequence. The 5′- and 3′- ends of each segment were amplified using the 5′/3′ rapid amplification of cDNA end kit (Clontech, Mountain View, CA, USA) following the guidelines of the manufacturer.

The initial complete genome was assembled and manually edited using ContigExpress software (ContigExpress LLC, New York, NY, USA). ORFs were predicted using online software ORF finder (https://www.ncbi.nlm.nih.gov/orffinder/). The putative protein functions of respective ORFs were then inferred by BLASTp in the non-redundant protein database. The nucleotide and amino acid sequence identity were analysed using the Clustal W method of MegAlign software (DNASTAR, Madison, WI, USA). Maximum likelihood phylogenetic analyses based on 10 ORFs nucleotide sequences were conducted using the General Time Reversible model using MEGA 7.0.14 software (www.megasoftware.net), and estimates based on bootstrap resampling were carried out with 1000 replicates.

### Electrophoresis of virus RNA

To compare the electrophoretic mobilities of the genomic segments of the isolate with representative ARV strains, the virus was propagated in DEFs and total RNA was extracted from the ultracentrifugation-concentrated virus suspension using TRIzol as described above. The extracted RNA was then treated with DNase (NEB, Ipswich, MA, USA) and S1 Nuclease (Promega, Madison, WI, USA) to remove DNA and single-stranded RNA. The duck reovirus isolate HC and ARV S1133 were propagated in DEF and chicken fibroblasts, respectively, as described^10^, and RNA was extracted and treated individually in the same way then used as controls. Treated RNA samples were subjected to electrophoresis on 10% sodium dodecyl sulfate-polyacrylamide gel (SDS-PAGE) at 180 V for 7 h and nucleic acid bands were stained using rapid sliver staining kit according to manufacture’s guideline (YEASEN Biotechnology Co., Ltd., Shanghai, China).

### Preparation of antiserum and serological assays

Antisera against the isolated virus, duck reovirus HC and ARV S1133 were separately prepared in 3-week-old SPF chickens (Merial-Vital Laboratory Animal Technology Co., Ltd., Beijing, China) by four immunisations at 2-week intervals with inactivated virus suspension. Sera were collected 12 days after the last immunisation. For the cross-reactivity assay, heat-inactivated homologous or heterologous antiserum was 2-fold serially diluted and mixed with an equal volume of the virus suspension containing 200 TCID_50_/0.1 mL (50% tissue culture infective dose). After incubation at 37 °C for 1 h, the serum-virus mixture was inoculated into five wells of DEF monolayers in a 96-well plate. Cytopathic effect (CPE) was recorded for 5 days. The neutralising antibody titre was the highest serum dilution that resulted in the cells being completely protected.

### Experimental infection of ducklings

One-day-old Pekin ducklings without maternal antibody against duck reovirus were purchased from Nankou Hatchery (Beijing Golden Star Duck Co., Ltd., Beijing, China). Ducklings were raised in negative pressured isolators with *ad libitum* access to feed and water. In experiment 1, twenty 2-day-old ducklings were divided into three groups and infected with the cell culture-prepared virus suspension as indicated in Table 5. Ducklings in group 1 were orally and intranasally infected with 0.5 mL cell culture suspension containing 7.35 × 10^4^ TCID_50_ of the isolate. Group 2 were subcutaneously infected with the same dose, and group 3 were raised separately as mock-infected controls. Clinical signs were recorded for 16 days to evaluate the pathogenicity of the isolate for ducklings, and cloacal swabs were collected as indicated for detection of virus shedding. On 9, 12 and 16 dpi, serum samples were collected for antibody detection using the virus neutralisation test as described above. To evaluate the *in vivo* infectivity of the isolate, ten ducklings at 12 days old were infected by oral and subcutaneous inoculation with the same dose virus as described above. Five ducks were sacrificed on both 3 and 5 dpi, respectively, and liver, spleen, caecal tonsil, thymus and bursa of Fabricius samples were collected for virus detection by RT-PCR and re-isolation. Five uninfected control ducklings were examined in the same way at each sampling point.

### Detection of viral RNA in tissue samples by RT-PCR

The viral RNA from tissue samples and cloacal swabs were extracted by viral RNA kit (Omega Bio-tek, Norcross, GA, USA) and converted to cDNA using a Reverse Transcription System (Promega, Madison, WI, USA) with specific primers following the manufacturer’s instructions. Specific primers based on the λC gene were designed and synthesised (Ych-4F: 5′-CTAAAGCTATTGACGTGGTGC-3′; Ych-4R: 5′-GGTAGTCCAACTGCATGTA G-3′). The length of RT-PCR product was 557 bp.

### Histopathological examination

Histological sections were routinely prepared from the bursa after samples were fixed in 10% neutral buffered formalin solution and paraffin embedded. The sections were stained with haematoxylin and eosin (HE). For apoptosis detection, TUNEL assay (dUTP nick end labelling) was conducted using an *In Situ* Cell Detection kit (Roche, Mannheim, Germany) following the manufacturer’s instructions.

### Ethics statement

Animal infection experiments were approved by the China Agricultural University Animal Ethics Committee, in accordance with the guidelines of the Review of Welfare and Ethics of Laboratory Animals approved by the Beijing Municipality Administration Office of Laboratory Animals. Experiments involving reovirus infections were conducted in the Biosafety Level 2 facilities in College of Veterinary Medicine, China Agricultural Unversity.

## Supplementary information

Supplementary material
