# Supplementary material for: Phenotypic and genetic characterisation of an emerging reovirus from Pekin ducks in China
Source: Sci Rep. 2019 May 23;9:7784. doi: 10.1038/s41598-019-44178-3 (PMC6533297; doi:10.1038/s41598-019-44178-3)
Supplement: Supplementary file 1 — Supplementary material [file 41598_2019_44178_MOESM1_ESM.pdf]

Phenotypic and genetic characterisation of an emerging reovirus from Pekin ducks in China

Yanxin Cao[1], Mengxu Sun[1], Jun Wang[1], Xueying Hu[2]*, Weiyong He[1] & Jingliang Su[1]*

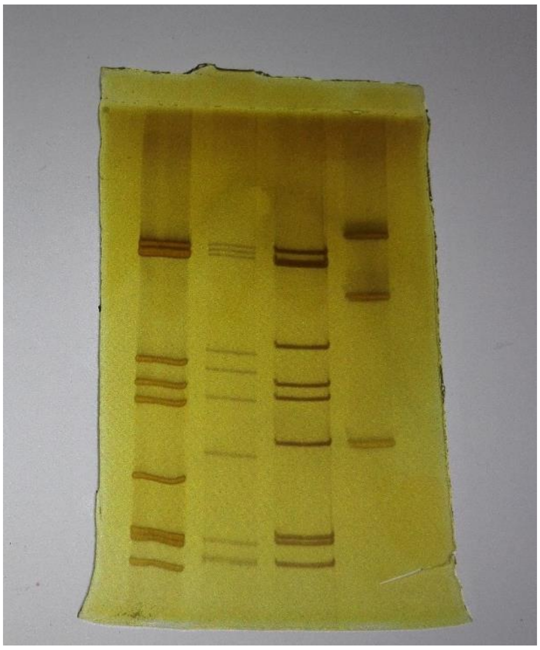

Figure S1. SDS-PAGE analysis of the genomic segment mobility of HC (a duck reovirus), Ych and S1133 (a chicken reovirus). Full-length gel of Figure 1c.

Table S1. Nucleotide and amino acid sequence identities of protein λA of the isolate Ych to representative members of avian reovirus (%)

Virus	1	2	3	4	5	6	7	8	9
1.S1133[a]	***	85.0	77.5	78.1	78.1	77.8	78.0	72.3	72.7
2.D1246[b]	96.6	***	77.4	77.9	77.6	78.2	77.9	72.5	72.1
3.ZJ00M[c]	94.7	94.8	***	94.4	86.8	86.2	87.1	71.4	71.6
4.HC[c]	94.3	94.4	99.0	***	86.4	86.5	86.9	71.8	72.0
5.03G[c]	94.4	94.3	98.0	97.7	***	89.9	89.1	72.4	72.2
6.ZJ2000M[d]	94.4	94.5	97.7	97.5	97.4	***	89.6	71.4	71.9
7.D20/99[d]	94.0	94.2	97.3	97.0	97.3	96.9	***	71.5	71.5
8.D2533/6/1-10[e]	83.8	84.0	83.6	83.4	83.6	83.5	83.5	***	94.1
9.Ych[e]	84.5	84.5	84.2	83.8	84.0	84.1	83.9	98.1	***

[a]chicken (S1133, KF741756.1) origin ARV; [b]turkey (D1246, KR997909.1) origin ARV; [c]"novel" waterfowl origin ARVs: Muscovy duck (ZJ00M, KF154110.1), Pekin duck (HC) and Goose (03G, JX145328.1); [d]"classical" waterfowl origin ARVs: Muscovy duck (ZJ2000M, KF306082.1) and Goose (D20/99, KF809662.1); [e]new emerge duck reovirus: Pekin duck (D2533/6/1-10, MH520085.1; Ych, MK173029); The amino acid sequence identities were highlighted with the gray background.

Table S2. Nucleotide and amino acid sequence identities of protein λB of the isolate Ych to representative members of avian reovirus (%)

Virus	1	2	3	4	5	6	7	8	9
1.S1133[a]	***	83.8	75.7	75.8	75.5	76.3	75.2	67.5	67.4
2.D1246[b]	95.6	***	76.0	75.7	75.8	76.1	75.3	66.5	66.8
3.ZJ00M[c]	91.3	91.2	***	98.0	87.9	88.5	88.5	65.9	66.0
4.HC[c]	91.3	91.2	99.3	***	87.8	88.4	88.4	66.2	66.0
5.03G[c]	91.4	91.0	97.5	97.3	***	93.4	91.6	66.9	66.5
6.ZJ2000M[d]	91.7	91.1	98.3	98.3	97.6	***	93.0	66.7	66.3
7.D20/99[d]	91.5	91.3	98.1	98.0	97.2	97.9	***	66.4	66.0
8.D2533/6/1-10[e]	75.6	74.8	75.1	75.0	75.2	75.3	74.8	***	91.7
9.Ych[e]	76.0	75.5	75.9	75.8	75.8	76.1	75.6	97.4	***

[a]chicken (S1133, KF741757.1) origin ARV; [b]turkey (D1246, KR997910.1) origin ARV; [c]"novel" waterfowl origin ARVs: Muscovy duck (ZJ00M, KF154111.1), Pekin duck (HC) and Goose (03G, JX145329.1); [d]"classical" waterfowl origin ARVs: Muscovy duck (ZJ2000M, KF306083.1) and Goose (D20/99, KF809663.1); [e]new emerge duck reovirus: Pekin duck (D2533/6/1-10, MH520086.1; Ych, MK173030); The amino acid sequence identities were highlighted with the gray background.

Table S3. Nucleotide and amino acid sequence identities of protein λC of the isolate Ych to representative members of avian reovirus (%)

Virus	1	2	3	4	5	6	7	8	9
1.S1133[a]	***	71.7	70.1	70.1	70.0	69.9	69.4	55.3	56.0
2.D1246[b]	82.0	***	70.3	70.3	70.1	70.3	69.5	55.6	55.7
3.ZJ00M[c]	79.5	79.7	***	98.4	96.8	79.9	78.9	56.7	56.2
4.HC[c]	79.4	79.6	99.7	***	97.5	80.3	79.2	56.8	56.2
5.03G[c]	79.2	79.1	98.6	98.8	***	80.4	79.4	56.9	56.4
6.ZJ2000M[d]	78.5	79.9	93.2	93.3	93.1	***	82.5	57.6	56.3
7.D20/99[d]	78.3	79.5	92.2	92.4	92.3	92.2	***	56.9	56.5
8.D2533/6/1-10[e]	55.1	54.4	56.2	56.2	56.1	55.8	55.8	***	87.7
9.Ych[e]	54.9	54.6	55.9	56.0	55.8	55.6	55.4	94.9	***

[a]chicken (S1133, KF741758.1) origin ARV; [b]turkey (D1246, KR997911.1) origin ARV; [c]"novel" waterfowl origin ARVs: Muscovy duck (ZJ00M, KF154112.1), Pekin duck (HC) and Goose (03G, JX145330.1); [d]"classical" waterfowl origin ARVs: Muscovy duck (ZJ2000M, KF306084.1) and Goose (D20/99, KF809664.1); [e]new emerge duck reovirus: Pekin duck (D2533/6/1-10, MH520087.1; Ych, MK173031); The amino acid sequence identities were highlighted with the gray background.

Table S4. Nucleotide and amino acid sequence identities of protein μA of the isolate Ych to representative members of avian reovirus (%)

Virus	1	2	3	4	5	6	7	8	9
1.S1133[a]	***	87.9	73.6	73.6	73.6	72.9	73.6	60.1	59.5
2.D1246[b]	95.4	***	74.1	74.1	74.1	72.7	73.2	60.3	60.3
3.ZJ00M[c]	86.6	86.4	***	97.9	96.4	81.0	79.9	60.2	60.5
4.HC[c]	86.2	86.4	98.4	***	97.0	81.0	80.4	60.3	60.5
5.03G[c]	86.2	86.1	97.0	97.8	***	80.9	80.1	60.4	60.7
6.ZJ2000M[d]	85.3	85.3	94.3	94.8	93.6	***	82.4	58.6	58.7
7.D20/99[d]	85.4	84.9	90.7	91.5	90.6	91.7	***	58.6	58.4
8.D2533/6/1-10[e]	59.6	59.2	59.2	59.4	59.7	59.2	58.6	***	93.4
9.Ych[e]	61.1	60.6	60.8	61.1	61.1	61.0	60.4	93.3	***

[a]chicken (S1133, KF741759.1) origin ARV; [b]turkey (D1246, KR997912.1) origin ARV; [c]"novel" waterfowl origin ARVs: Muscovy duck (ZJ00M, KF154113.1), Pekin duck (HC) and Goose (03G, JX145331.1); [d]"classical" waterfowl origin ARVs: Muscovy duck (ZJ2000M, KF306085.1) and Goose (D20/99, KF809665.1); [e]new emerge duck reovirus: Pekin duck (D2533/6/1-10, MH520088.1; Ych, MK173032); The amino acid sequence identities were highlighted with the gray background.

Table S5. Nucleotide and amino acid sequence identities of protein μB of the isolate Ych to representative members of avian reovirus (%)

Virus	1	2	3	4	5	6	7	8	9
1.S1133[a]	***	75.5	76.7	76.5	76.2	69.3	68.9	64.8	64.8
2.D1246[b]	88.0	***	76.5	76.1	75.7	67.4	67.1	64.3	65.2
3.ZJ00M[c]	89.6	89.9	***	98.2	87.9	67.6	67.8	65.2	65.8
4.HC[c]	89.5	89.8	99.0	***	88.4	67.4	67.7	65.1	65.7
5.03G[c]	88.9	89.4	96.0	96.2	***	67.7	67.5	65.9	65.9
6.ZJ2000M[d]	75.6	76.1	76.3	76.5	75.6	***	92.5	62.1	62.8
7.D20/99[d]	75.2	75.9	76.3	76.2	75.3	96.9	***	61.9	62.5
8.D2533/6/1-10[e]	70.3	72.3	70.9	71.2	71.7	67.2	66.6	***	89.6
9.Ych[e]	70.0	72.1	71.2	71.5	72.0	66.9	66.5	98.5	***

[a]chicken (S1133, KF741760.1) origin ARV; [b]turkey (D1246, KR997913.1) origin ARV; [c]"novel" waterfowl origin ARVs: Muscovy duck (ZJ00M, KF154114.1), Pekin duck (HC) and Goose (03G, JX145332.1); [d]"classical" waterfowl origin ARVs: Muscovy duck (ZJ2000M, KF306086.1) and Goose (D20/99, KF809666.1); [e]new emerge duck reovirus: Pekin duck (D2533/6/1-10, MH520089.1; Ych, MK173033); The amino acid sequence identities were highlighted with the gray background.

Table S6. Nucleotide and amino acid sequence identities of protein μNS of the isolate Ych to representative members of avian reovirus (%)

Virus	1	2	3	4	5	6	7	8	9
1.S1133[a]	***	88.6	71.8	71.5	72.0	71.6	72.5	59.0	58.6
2.D1246[b]	94.2	***	72.0	71.9	71.9	71.6	71.2	58.5	58.2
3.ZJ00M[c]	80.5	80.8	***	98.1	94.8	86.4	79.3	58.3	57.6
4.HC[c]	81.0	81.1	98.6	***	95.0	86.6	79.4	58.1	57.8
5.03G[c]	80.7	80.8	96.4	97.0	***	87.0	78.9	58.2	57.8
6.ZJ2000M[d]	80.5	81.0	93.7	94.0	93.9	***	78.7	58.8	58.2
7.D20/99[d]	80.7	80.7	90.1	90.9	90.1	89.9	***	57.5	58.5
8.D2533/6/1-10[e]	56.5	57.3	56.0	56.6	56.5	55.8	57.1	***	92.1
9.Ych[e]	56.0	56.5	56.0	56.6	56.5	55.8	57.1	95.8	***

[a]chicken (S1133, KF741761.1) origin ARV; [b]turkey (D1246, KR997914.1) origin ARV; [c]"novel" waterfowl origin ARVs: Muscovy duck (ZJ00M, KF154115.1), Pekin duck (HC) and Goose (03G, JX145333.1); [d]"classical" waterfowl origin ARVs: Muscovy duck (ZJ2000M, KF306087.1) and Goose (D20/99, KF809667.1); [e]new emerge duck reovirus: Pekin duck (D2533/6/1-10, MH520090.1; Ych, MK173034); The amino acid sequence identities were highlighted with the gray background.

Table S7. Nucleotide and amino acid sequence identities of protein σC of the isolate Ych to representative members of avian reovirus (%)

Virus	1	2	3	4	5	6	7	8	9
1.S1133[a]	***	61.0	39.1	39.3	39.0	35.6	34.9	42.5	43.6
2.D1246[b]	57.2	***	40.0	40.6	40.3	36.5	36.5	41.1	40.7
3.ZJ00M[c]	28.6	27.0	***	97.3	97.4	52.0	50.7	41.7	40.7
4.HC[c]	28.6	27.0	97.5	***	97.8	51.7	51.2	42.0	40.9
5.03G[c]	28.3	27.4	97.5	98.8	***	52.4	51.3	41.4	40.4
6.ZJ2000M[d]	25.2	21.1	41.5	41.5	41.9	***	90.7	37.2	37.5
7.D20/99[d]	25.9	22.6	41.1	41.1	41.5	91.9	***	37.2	37.3
8.D2533/6/1-10[e]	21.6	21.6	27.6	27.0	27.0	24.4	24.8	***	85.5
9.Ych[e]	22.2	22.2	27.3	26.7	26.7	25.2	24.8	91.7	***

[a]chicken (S1133, KF741762.1) origin ARV; [b]turkey (D1246, KR997915.1) origin ARV; [c]"novel" waterfowl origin ARVs: Muscovy duck (ZJ00M, KF154116.1), Pekin duck (HC) and Goose (03G, JX145334.1); [d]"classical" waterfowl origin ARVs: Muscovy duck (ZJ2000M, KF306091.1) and Goose (D20/99, KF809668.1); [e]new emerge duck reovirus: Pekin duck (D2533/6/1-10, MH520091.1; Ych, MK173035); The amino acid sequence identities were highlighted with the gray background.

Table S8. Nucleotide and amino acid sequence identities of protein σA of the isolate Ych to representative members of avian reovirus (%)

Virus	1	2	3	4	5	6	7	8	9
1.S1133[a]	***	91.3	77.5	77.1	77.0	76.4	76.4	63.5	63.6
2.D1246[b]	96.9	***	77.5	77.9	77.4	77.1	77.3	62.5	63.7
3.ZJ00M[c]	91.6	91.8	***	88.7	88.8	89.8	86.6	63.2	63.8
4.HC[c]	91.4	91.6	98.3	***	97.0	95.3	87.8	64.4	64.3
5.03G[c]	90.9	91.1	97.6	99.3	***	95.4	87.8	64.0	64.1
6.ZJ2000M[d]	91.1	91.4	98.6	99.3	98.6	***	88.8	64.4	64.3
7.D20/99[d]	90.4	90.9	96.4	96.6	95.9	96.9	***	63.9	63.9
8.D2533/6/1-10[e]	66.7	66.7	66.4	67.1	66.9	66.9	66.9	***	89.6
9.Ych[e]	66.9	66.9	66.7	67.4	67.1	67.1	67.1	96.4	***

[a]chicken (S1133, KF741763.1) origin ARV; [b]turkey (D1246, KR997916.1) origin ARV; [c]"novel" waterfowl origin ARVs: Muscovy duck (ZJ00M, KF154117.1), Pekin duck (HC) and Goose (03G, JX145335.1); [d]"classical" waterfowl origin ARVs: Muscovy duck (ZJ2000M, KF306088.1) and Goose (D20/99, KF809669.1); [e]new emerge duck reovirus: Pekin duck (D2533/6/1-10, MH520092.1; Ych, MK173036); The amino acid sequence identities were highlighted with the gray background.

Table S9. Nucleotide and amino acid sequence identities of protein σB of the isolate Ych to representative members of avian reovirus (%)

Virus	1	2	3	4	5	6	7	8	9
1.S1133[a]	***	71.6	66.0	66.5	66.0	60.7	60.2	56.2	55.8
2.D1246[b]	77.7	***	66.3	66.7	66.6	61.1	61.1	58.4	58.7
3.ZJ00M[c]	69.0	68.5	***	98.0	94.8	66.6	65.6	62.3	61.2
4.HC[c]	69.3	69.8	97.3	***	95.9	66.6	65.8	62.2	61.1
5.03G[c]	69.0	70.1	94.6	96.5	***	66.6	66.0	61.4	60.8
6.ZJ2000M[d]	62.0	63.3	69.3	70.4	69.6	***	93.2	58.8	59.2
7.D20/99[d]	60.6	62.0	68.8	69.8	69.3	94.0	***	58.7	59.1
8.D2533/6/1-10[e]	53.3	55.2	58.7	59.8	59.8	55.7	56.0	***	91.8
9.Ych[e]	52.4	55.4	59.2	60.3	60.3	56.2	56.5	97.6	***

[a]chicken (S1133, KF741764.1) origin ARV; [b]turkey (D1246, KR997917.1) origin ARV; [c]"novel" waterfowl origin ARVs: Muscovy duck (ZJ00M, KF154118.1), Pekin duck (HC) and Goose (03G, JX145336.1); [d]"classical" waterfowl origin ARVs: Muscovy duck (ZJ2000M, KF306089.1) and Goose (D20/99, KF809670.1); [e]new emerge duck reovirus: Pekin duck (D2533/6/1-10, MH520093.1; Ych, MK173037); The amino acid sequence identities were highlighted with the gray background.

Table S10. Nucleotide and amino acid sequence identities of protein σNS of the isolate Ych to representative members of avian reovirus (%)

Virus	1	2	3	4	5	6	7	8	9
1.S1133[a]	***	79.5	78.3	79.9	79.9	79.5	79.7	62.2	62.2
2.D1246[b]	92.3	***	77.5	77.4	77.6	77.3	77.0	61.0	61.6
3.ZJ00M[c]	90.8	90.1	***	94.6	94.3	85.1	91.8	61.3	61.0
4.HC[c]	91.3	90.4	98.4	***	94.7	85.3	91.5	60.9	61.3
5.03G[c]	91.8	91.0	97.8	98.9	***	85.3	92.4	61.1	60.9
6.ZJ2000M[d]	92.7	91.2	97.3	97.3	97.3	***	85.1	62.0	62.0
7.D20/99[d]	92.1	90.7	96.7	97.3	97.8	97.8	***	61.1	61.3
8.D2533/6/1-10[e]	65.5	63.8	63.6	64.1	63.9	64.4	63.9	***	90.3
9.Ych[e]	64.9	63.3	63.3	63.9	63.6	64.1	63.3	97.0	***

[a]chicken (S1133, KF741765.1) origin ARV; [b]turkey (D1246, KR997918.1) origin ARV; [c]"novel" waterfowl origin ARVs: Muscovy duck (ZJ00M, KF154119.1), Pekin duck (HC) and Goose (03G, JX145337.1); [d]"classical" waterfowl origin ARVs: Muscovy duck (ZJ2000M, KF306090.1) and Goose (D20/99, KF809671.1); [e]new emerge duck reovirus: Pekin duck (D2533/6/1-10, MH520094.1; Ych, MK173038); The amino acid sequence identities were highlighted with the gray background.
